# Uncovering a Dynamic Feature of the Transcriptional Regulatory Network for Anterior-Posterior Patterning in the *Drosophila* Embryo

**DOI:** 10.1371/journal.pone.0062641

**Published:** 2013-04-30

**Authors:** Junbo Liu, Jun Ma

**Affiliations:** 1 Division of Biomedical Informatics, Cincinnati Children’s Research Foundation, Cincinnati, Ohio, United States of America; 2 Division of Developmental Biology, Cincinnati Children’s Research Foundation, Cincinnati, Ohio, United States of America; University College London, United Kingdom

## Abstract

Anterior-posterior (AP) patterning in the *Drosophila* embryo is dependent on the Bicoid (Bcd) morphogen gradient. However, most target genes of Bcd also require additional inputs to establish their expression domains, reflective of the operation of a cross-regulatory network and contributions of other maternal signals. This is in contrast to *hunchback* (*hb*), which has an anterior expression domain driven by an enhancer that appears to respond primarily to the Bcd input. To gain a better understanding of the regulatory logic of the AP patterning network, we perform quantitative studies that specifically investigate the dynamics of *hb* transcription during development. We show that Bcd-dependent *hb* transcription, monitored by the intron-containing nascent transcripts near the P2 promoter, is turned off quickly–on the order of a few minutes–upon entering the interphase of nuclear cycle 14A. This shutdown contrasts with earlier cycles during which active *hb* transcription can persist until the moment when the nucleus enters mitosis. The shutdown takes place at a time when the nuclear Bcd gradient profile in the embryo remains largely intact, suggesting that this is a process likely subject to control of a currently unknown regulatory mechanism. We suggest that this dynamic feature offers a window of opportunity for *hb* to faithfully interpret, and directly benefit from, Bcd gradient properties, including its scaling properties, to help craft a robust AP patterning outcome.

## Introduction

Transcriptional regulatory networks play critical roles in embryonic patterning [1]–[6]. In *Drosophila*, embryonic patterning along the anterior-posterior (AP) axis is initiated by the expression of gap genes in response to the morphogen gradient Bicoid (Bcd) and an extensive cross-regulatory network [4], [7]–[12]. The *Drosophila* AP patterning network represents an important paradigm for mechanistic understanding of how precise patterning is achieved [6], [10], [13]. One distinct aspect of patterning precision is related to scaling, the formation of patterns that are proportional to, or scaled with, the length of the embryo [14]–[19]. Scaling is a fundamental feature of embryonic development [6], [20]–[22]. Modeling studies show that cross-regulation among gap genes can achieve precise and scaled patterning outcome [13], [23]. In addition, the Bcd gradient itself exhibits scaling properties within a species [15], [17], suggesting that developmental precision may also benefit directly from Bcd if the scaling properties of this morphogen gradient can be directly and faithfully interpreted by its target gene(s) in controlling their expression patterns.

**Figure 1:**
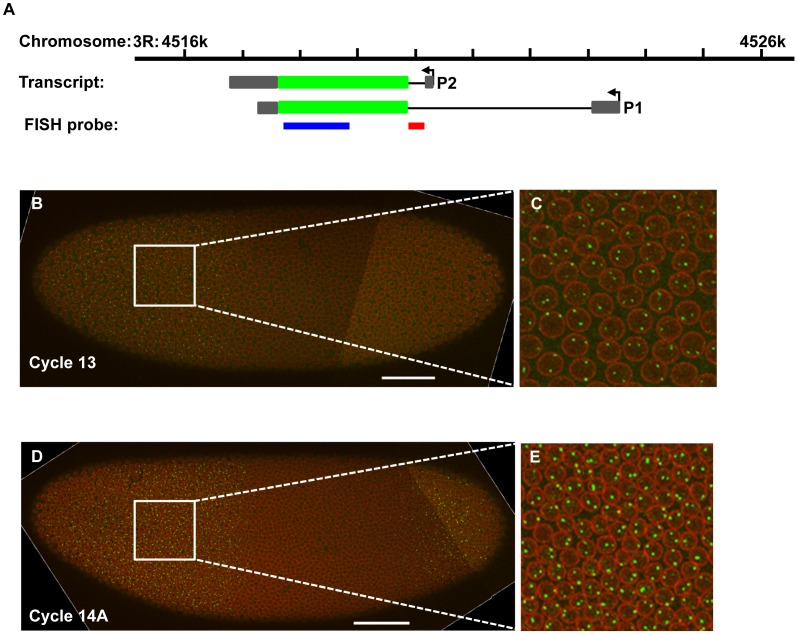
Intron staining detecting nascent transcripts near the *hb* P2 promoter. (**A**) Shown is a schematic diagram of the *hb* locus and the locations of the probes used in this study. P1 and P2 are two promoters for *hb* transcription leading to two types of transcripts, where exons are represented by boxes with coding regions filled in green and non-coding in grey; introns are represented by thin lines. The heavy blue line represents the probe for detecting the mature *hb* mRNA and the heavy red line represents the intronic probe. (**B**) Shown is a merged confocal image of an embryo at cycle 13. The detected nuclear envelope is shown in red and the nascent *hb* transcripts detected (with an intronic probe) as intron dots are in green. Scale bar, 50 µm. (**C**) Shown is a magnified view of a section of the expression region from panel **A**. (**E** and **F**) Shown are a merged confocal image (**E**) of an embryo in cycle 14A and a magnified view (**F**) of a section of the expression region from panel **C**.

The gap gene *hunchback* (*hb*), a target of Bcd, has been subject to extensive investigations [8], [14], [24]–[27]. It has an expression domain in the anterior half of the embryo with a precise and scaled boundary position [14]. Importantly, scaling properties near the middle of the embryo (along the AP axis) are greatly influential to the patterning landscape along the entire AP length [19], [28]–[30]. The *hb* gene contains two promoters and three known enhancers that together orchestrate the dynamic expression patterns during development [24], [31]. The Bcd-dependent *hb* transcripts, expressed from the proximal P2 promoter, are detectable as early as nuclear cycle 9, which continue to accumulate as embryo development progresses [26]. By the mid of nuclear cycle 14A (on the order of ∼20–30 min into the interphase), the primary pattern of the mature *hb* mRNA peaks in level and then begins to decay. This decaying primary pattern is concurrently replaced by an emerging secondary pattern, which consists primarily of a stripe at parasegment 4 (PS4) and a stripe at the posterior of the embryo [8], [31]–[35]. Expression of *hb* at both PS4 and posterior domains is independent of Bcd as a direct input and is transcribed from both P2 and the distal promoter P1 ([24], [31]; see Fig. 1A for a schematic diagram).

The posterior boundary of the anterior *hb* expression domain is thus from two distinct expression domains that have overlapping posterior boundaries, the Bcd-dependent primary *hb* domain and the Bcd-independent PS4 stripe [24], [32]. This suggests that both Bcd input and cross-regulatory inputs could have a role in controlling the position and/or precision of the *hb* expression boundary. But the exact contributions of these inputs–and their relationship–in controlling *hb* boundary formation are not well understood. On the one hand, experimental perturbations that alter Bcd gradient properties, including its scaling/precision and concentration properties, can lead to corresponding alterations in the *hb* expression pattern [17], [36]–[38]. Unlike the posterior boundaries of other gap genes such as *Kruppel* (*Kr)* and *knirps* (*kni)*, the *hb* boundary remains relatively stable during development [4], [39], [40]. In addition, this boundary does not significantly change upon mutating *Kr* or *kni* individually [13], [14], [41]. These findings suggest that the Bcd input plays a key role in determining the properties of *hb* expression boundary [28]. On the other hand, simultaneous loss of both *Kr* and *kni* can significantly alter *hb* expression [10], [13], [41], [42], indicating a role of cross-regulatory mechanisms in controlling *hb* expression. In addition, the widely-accepted topology of the gap cross-regulatory network can achieve a precise and scaled *hb* expression boundary despite a noisy Bcd gradient input that lacks scaling properties [6], [10], [13].

Illuminating earlier studies reported findings that offered tantalizing hints on the complexity and regulatory logic of *hb* expression, pointing to a potential direction for dissecting the contributions of Bcd and cross-regulatory inputs on *hb* boundary formation. In particular, while the PS4 *hb* stripe is inhibited by ectopic Kni expression at mid cycle 14A, the primary *hb* domain that is on its way of decay does not appear to respond to this particular manipulation [41]. In addition, unlike the expression patterns of other gap genes such as *Kr* and *kni*, which encode regulators of the PS4 stripe [24], the primary *hb* domain fades away quickly during cycle 14A [33], [39], [43]. These results raise interesting questions about the dynamics and regulatory mechanisms of gap gene transcription. Specifically, what is the dynamics of *hb* transcription near its P2 promoter during cycle 14A at the transition from the primary to secondary patterns? We reasoned that, since this transition marks the concurrent appearance of the Bcd-independent PS4 stripe and decay of the Bcd-dependent primary domain that have overlapping boundaries [24], [41], characterization of the dynamic properties during this period may benefit our deeper level understanding of the contributions of both Bcd input and cross-regulatory inputs to *hb* boundary formation and, by extension, the regulatory logic of the entire AP patterning network. Here we describe experiments to specifically investigate the dynamics of *hb* transcription near the P2 promoter during cycle 14A. P2 is used for *hb* transcription to form both patterns ([31]; Fig. 1A). Our results reveal a previously-uncharacterized feature of *hb* transcription, namely, active *hb* transcription (for its primary domain) near the P2 promoter becomes shut down quickly upon entering the cycle 14A interphase. We suggest that this shutdown is an important operating feature of the AP patterning network, which offers a window of opportunity for *hb* to directly and faithfully interpret the Bcd gradient input and contribute to the precise and scaled patterning outcome.

## Materials and Methods

### Embryo Staining, Imaging and Data Analysis

All embryos (0–4 hours) were from *w^1118^* and were collected at 25°C. Quantitative immunostaining for Bcd (anti-Bcd antibodies; Santa Cruz Biotechnology) in embryos, imaging with ApoTome microscope and intensity measurements were described previously [38]. Quantitative FISH detecting mature *hb* mRNA, imaging with ApoTome microscope and data analysis were as reported previously [38]. For extracting data along the AP axis, each embryo is divided into 50 equal-size bins as described previously [17]. *hb_plat_* was defined as the mean *hb* intensity at the plateau region and calculated as follows. For each embryo, 5 consecutive bins located 0.1 *x/L* (fractional embryo length) anterior to its *hb* expression boundary position (*x_hb_*, defined as the AP position at which the detected mRNA intensity is at half maximal) were grouped together and the mean intensity value of this group was defined as *hb_plat_*. Thus, *hb_plat_* is devoid of both PS4 and the variable anterior stripe [33].

Quantitative intron staining to detect nascent *hb* or *Kr* transcripts as intron dots in embryos was performed as follows. Digoxigenin (Roche)-labeled antisense RNA probes targeting the *hb* or *Kr* introns were prepared and intron staining was performed as described previously [44]. The primers used for amplifying *hb* intronic sequence have been described in a previous publication [44]. To amplify *Kr* intronic sequence, the following pair of primers was used: 5′-CCGGAATTCTTCTCATTATTAAACAGTCC-3′ and 5′-CGCGGATCCTAAGAGCA ACGGCTTAAAAGAG-3′. To maximize the number of intron dots detected in whole mount embryos, we captured high resolution (1024 × 1024; 0.31 µm/pixel) confocal images for embryos that had been flattened as described before [44]. Briefly, a zoom scale of 0.7 was selected to have an imaging field covering the entire anterior *hb* expression domain. We generally took 8–10 *z*-sections (covering 4–5 µm along the *z* axis) to capture all the *hb* intron dots in each nucleus [45]. Embryo size measurements required stitching these images to a different set of images (under same imaging setting) that captured the posterior part of the embryo. We note that stitched images, where stitches can be visible, were only used for measuring embryo length. We ensured to capture, in a single set of images without stitching, all the *hb* intron dots in the anterior region of an individual embryo. For each embryo, which was co-stained with wheat germ agglutinin (WGA) with Alexa Fluor® 555 conjugate, a separate image was captured on the midsagittal plane for time class division (see below). In our quantitative analysis, threshold settings for intron dot detection were as described previously [26]. Specifically, the pixel number threshold (which is defined as the minimum number of connected pixels within a cluster of intensity signals) was set to be ≥ 3 and the intensity threshold for an individual embryo was set to be the lower limit at which no nuclei in the *hb* or *Kr* expression domain had more than two intron dots detected. As shown before [26], [44], the number of *hb* intron dots in individual nuclei have rarely exceeded 2, suggesting that either the active *hb* transcription at nuclear cycle 14A takes place predominantly before DNA replication or the sister chromatids are tightly connected at the *hb* locus. The mean *hb* intron dot number per nucleus at the plateau region, *ρ_plat_*, was calculated for each embryo in a manner identical to that for *hb_plat_* (above). Specifically, each embryo was divided into 50 equal-size bins along the AP axis. The *ρ* value for each bin was calculated and plotted against *x/L* to establish a profile. The posterior boundary position of the anterior domain (*x_hb_*), defined as the AP position at which half-maximal *ρ* was detected, was then calculated as described previously [17]. *ρ_plat_* was defined as the mean *ρ* value at the plateau (with regard to AP position) and calculated by averaging the values from 5 consecutive bins located 0.1 *x/L* anterior to *x_hb_*. For our *Kr* intron dot data, we used a parameter *ρ_peak_* to quantify the peak level of *Kr* intron dot number per nucleus. This value was calculated for each embryo by averaging the *ρ* values from three consecutive bins with the center bin being at the peak position. The peak position is defined as the AP position within the central expression stripe where the *ρ* value is at maximum for an embryo. Image processing and quantitative analysis were performed through the Matlab software (MathWorks). Computer codes and experimental data reported in this work are available upon request.

### Nuclear Cycles and Time Class Division for Embryos at Early Cycle 14A

**Figure 2:**
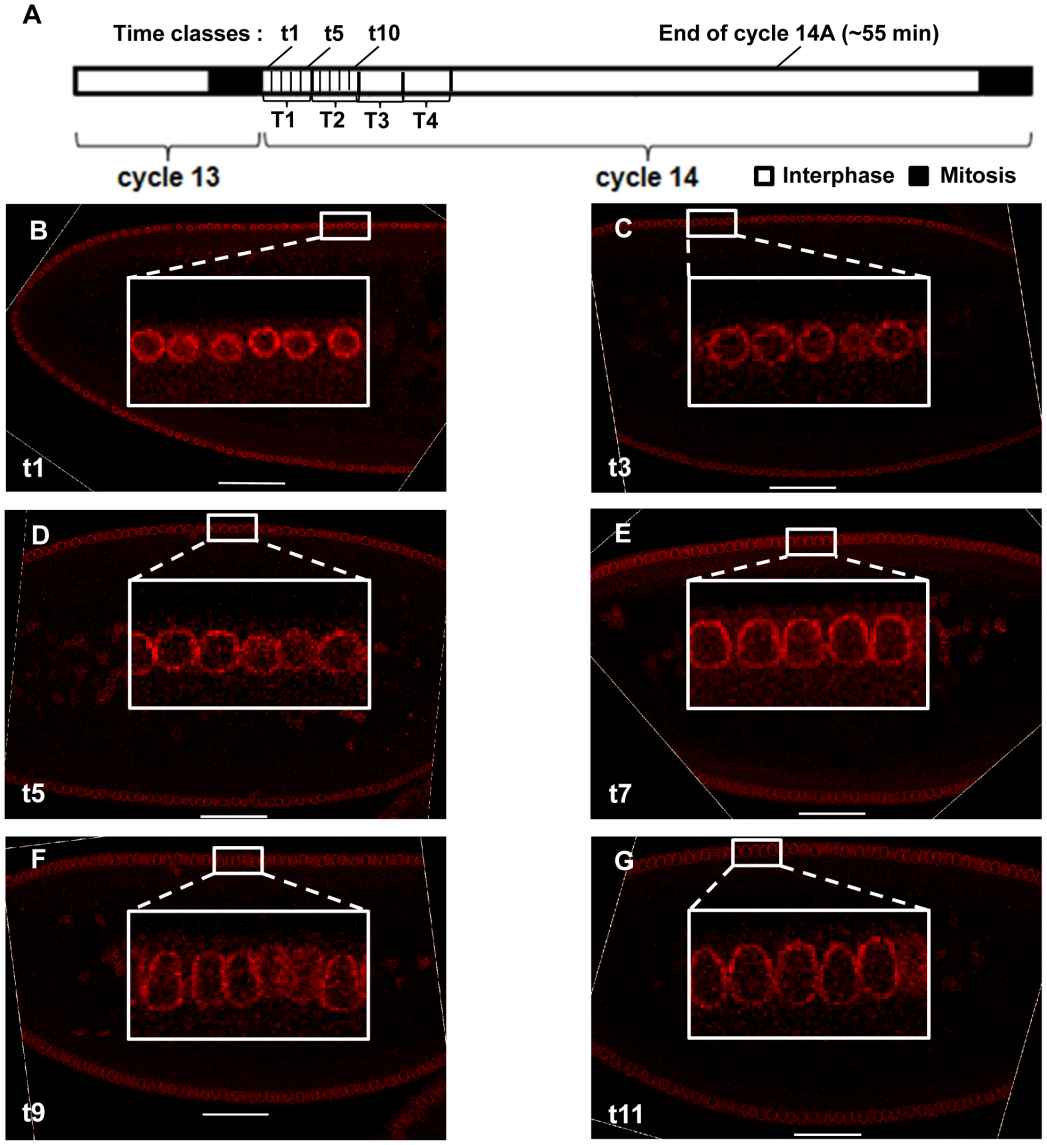
Progressive change in nuclear morphology during early cycle 14A. (A) Shown is a schematic diagram depicting the relationship between the t and T time classes at cycle 14A and other time events. (B–G) Shown are midsagittal images of embryos time classes of t1 (B), t3 (C), t5 (D), t7 (E), t9 (F), and t11 (G), respectively. Here the nuclear envelope is shown in red, with the inserts exhibiting magnified views. Images shown here were adjusted for presentational purposes only. Scale bar, 50 µm.

The number of nuclei on the dorsal side of an embryo’s midsagittal image was used to determine its nuclear cycle. Typically, embryos at nuclear cycle 13 have ∼55–65 identifiable nuclei that are round in shape. Embryos at nuclear cycle 14A have ∼70–80 identifiable nuclei, which are round in shape initially and become progressively elongated (along the apical-basal axis) as a function of developmental time [46], [47]. As an independent method for verification, we also counted the total number of nuclei from the projected “surface” image for each embryo. There are ∼1500, 3000 and 6000 nuclei on the entire periphery of embryos at cycles 12, 13 and 14A, respectively [47]. Images capturing one surface of the flattened embryos are expected to have ∼half of the total number of nuclei. In our analysis, cycle 13 embryos had between 787 and 1296 nuclei captured, and cycle 14A embryos had between 1530 and 2463 nuclei captured. To divide embryos at cycle 14A into time classes for intron dot analysis, we first calculated the mean nuclear height for each embryo based on its midsagittal image (see above and Fig. 2). Nuclear height was defined as the distance between the outer edges of the detected membrane on the apical and basal sides of the nucleus [46]. Nuclear heights were measured manually using the Overlay function in the Zeiss LSM image browser. For each embryo, we manually selected nuclei near the “mid-lateral” regions for height measurements. These selected nuclei have well resolved staining for the nuclear envelope. We typically measured 10 nuclei (5 from each side) to obtain the average nuclear height for an embryo. All embryos at early cycle 14A were ranked based on their measured average nuclear heights. Time class divisions for these embryos were then made from this simple ranking using cutoff points according to established fitting [46]. Our estimates based on the distributions of the nuclear heights in the time classes (Fig. S1) suggest they have an uncertainty in the range of ∼0.6–0.9 min. These time classes thus represent reliable temporal rankings of the embryos (see Fig. S1 legend for more information).

## Results

### Experimental Design

Previous studies that investigate the dynamic properties of *hb* expression have primarily focused on mature *hb* mRNA or Hb protein [4], [39], [43] and did not have a fine resolution of time. Mature transcripts from P1 and P2 have been detected separately during cycle 14A embryos with specific probes, but dynamic information at a fine temporal resolution is unavailable [31], [48]. To gain a better understanding of the dynamics of *hb* transcription during development, we performed quantitative fluorescence *in situ* hybridization (FISH) that detects either the mature *hb* mRNA or its nascent transcripts (see Materials and Methods). We used the “standard” FISH to monitor the mature mRNA level in the cytoplasm and an “intron staining” (through the use of a probe against the 283-nucleotide *hb* intron RNA sequence) to detect its nascent transcripts inside the nucleus [44], [49]–[51]. The *hb* intron detected by our probe is located relatively close (145 bp) to the Bcd-responsive P2 promoter [31]. In addition, intron sequences are thought to be removed quickly from the nascent transcripts after RNA polymerase II (RNAP) has transcribed them [51]. Thus, unlike the mature *hb* mRNA, which represents time-averaged products of transcription, the detected intron sequence-containing nascent transcripts inside the nucleus monitor more closely (but still not exactly) the actual dynamics of *hb* transcription initiation/elongation from the P2 promoter as a function of time. In our experiments, the nascent transcripts are detected as discrete fluorescent dots, referred to “intron dots” [44], [50]. These intron dots thus represent snapshots of individual copies of the *hb* gene undergoing active transcription near its P2 promoter (Fig. 1B–E). Importantly, the detected *hb* intron dots form an expression pattern along the AP axis resembling the well documented mature *hb* mRNA pattern ([44]; see also below), documenting the sensitivity and reliability of the technique.

Upon entering the cycle 14A interphase, the height of the nuclei (which is the apical-to-basal distance between the outer edges of the detected membrane [46]) increases nearly linearly as a function of time doubling in <15 min [46], providing a sensitive means for dividing embryos into time classes (Fig. S1; see Materials and Methods). In our current study, the time class did not go beyond ∼20 min into the cycle 14A interphase because later times are not critical to our analysis and, furthermore, at later times the nuclear height is no longer a reliable measure of time [39], [46]. In our study, we grouped embryos into time classes based on the simple ranking of their mean nuclear heights measured from midsagittal images of individual embryos. Two temporal scales were used in our dynamic analysis with an estimated mean time difference of either ∼1 or ∼5 min, referred to t and T time classes, respectively (see Fig. 2A for a schematic diagram showing the estimated locations of these time classes in relation to each other and to other relevant time events). Figs. 2B–G show midsegittal images of representative embryos that belong to t1, t3, t5, t7, t9 and t11, illustrating the progressive change in nuclear morphology (see also Fig. S1 for distributions of nuclear heights in the t time classes). In all of our quantitative staining, we did not use methods to amplify signals and ensured that all images were captured within a linear range without signal saturation (see [15], [17], [38] for further information about linearity between molecule number and fluorescence signal).

### Distinct Dynamic Properties of Mature *hb* mRNA and *hb* Nascent Transcripts in Cycle 14A Embryos

Fig. S2 shows images of four representative embryos that belong to time classes T1–T4, respectively, following the dynamic properties of the primary pattern of Bcd-driven mature *hb* mRNA. These results show that, consistent with previous reports [43], the mature *hb* mRNA level remains relatively stable during this time period. In the embryo belonging to class T3 (estimated to be ∼10–15 min into this cycle), the *hb* mRNA level in the anterior reaches its peak, and begins to drop gradually in embryos later in time. This relative stability of mature *hb* mRNA profile during cycle 14A is in comparison with the transcription dynamics that we discuss below; compared with mature mRNA patterns of other gap genes, the primary pattern of mature *hb* mRNA is known to be less stable [43].

**Figure 3:**
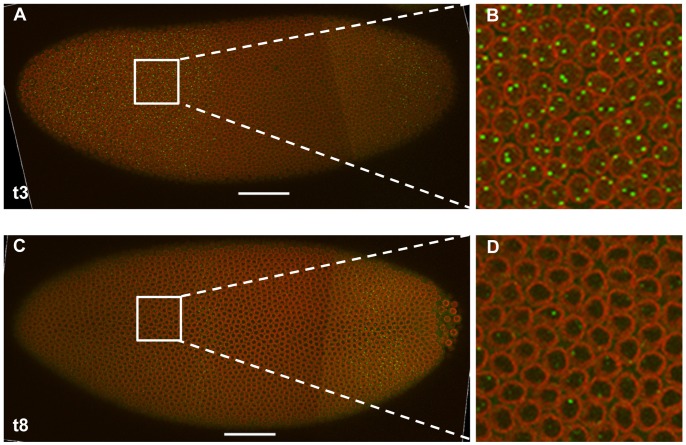
*hb* nascent transcripts decay quickly at early nuclear cycle 14A. (**A**) Shown is a merged confocal image on the “surface” of an embryo at t3. (**B**) shown is a magnified view of panel A. Nascent transcripts were detected by an intronic probe. Scale bar, 50 µm. (**C** and **D**) Same as **A** and **B**, except this embryo is at the time class of t8.

One of our initial observations in evaluating our intron-stained embryos was that only a small fraction of embryos at cycle 14A had bright intron dots in the anterior detected by confocal imaging. These results are consistent with the possibility [49] that active *hb* transcription may be significantly delayed upon exiting mitosis, thus only detectable in those embryos that have progressed well into the cycle 14A interphase and have overcome this delay. Alternatively, it is possible that active *hb* transcription begins quickly after mitosis but becomes shut off quickly, thus readily detectable only in those embryos that have not yet progressed far into cycle 14A interphase. To differentiate between these possibilities and better follow the *hb* transcription dynamics as a function of time, we began, for all of our subsequent experiments, to capture images on the midsagittal section of each stained embryo in addition to the “surface” images that capture the intron dots (see above). Fig. 3 shows the surface images from two representative embryos belonging to the time classes of t3 and t8. While the embryo at t3 exhibits a strong pattern of *hb* intron staining in the anterior, the embryo at t8 has a diminishing number of bright fluorescent dots. These results suggest that Bcd-dependent active *hb* transcription near the P2 promoter, as monitored by the intron sequence-containing nascent transcripts, takes place efficiently only in cycle 14A embryos prior to time class t8 (see Fig. 2A for a reference diagram of relevant time events). Under confocal microscopy, we also examined visually embryos at times later than t14 based on temporal markers such as nuclear morphology. For those “really late” embryos, virtually no bright dots were detectable in the anterior, suggesting that *hb* transcription did not become “re-activated” in cycle 14A interphase.

### Quantitative Analysis of *hb* Transcription Dynamics at Cycle 14A Reveals a Quick Shutdown

**Figure 4:**
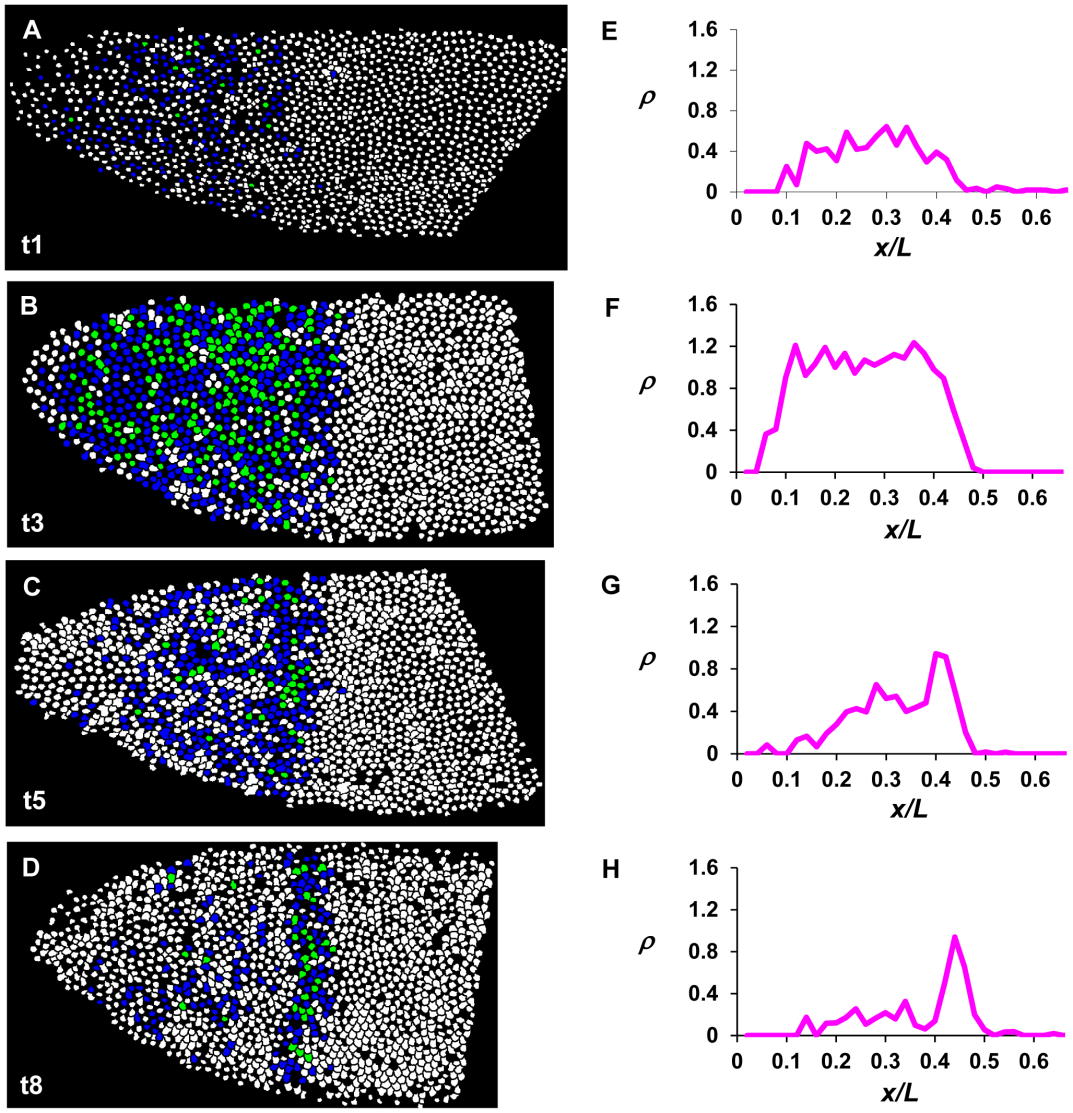
Quantitative analysis of active *hb* transcription at cycle 14A. (**A–D**) Shown are diagrams of embryos at the time classes of t1, t3, t5 and t8, respectively. Here each nucleus detected from the confocal images is shown and assigned a color based on the number of detected *hb* intron dots (nascent transcripts were detected by an intronic probe): white, no intron dots; blue, one dot; and green, two dots. (**E–H**) Shown are the ρ profiles as a function of *x/L* from panels **A–D**, respectively. ρ is defined as the mean intron dot number per nucleus.

**Figure 5:**
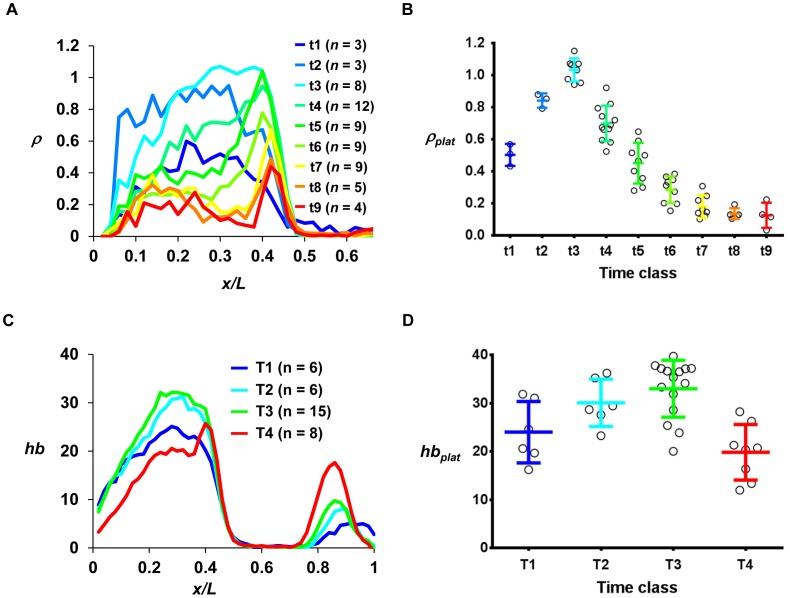
Dynamics of Bcd-dependent transcription at cycle 14A. (**A**) Shown are the mean *ρ* profiles as a function of *x/L* at early nuclear cycle 14A. Each color represents a time class; the adjacent t time classes differ by ∼1 min (see Fig. 2A). See Fig. S1 for additional information about the time classes shown here. For the sake of clarity, standard deviations are not shown in this figure. See Fig. S3 for standard deviations. (**B**) Shown are *ρ_plat_* values for individual embryos from different time classes. The mean and standard deviation are: 0.50±0.07, 0.84±0.05, 1.03±0.07, 0.70±0.11, 0.45±0.13, 0.29±0.08, 0.18±0.07, 0.14±0.03, 0.13±0.08 for t1 to t9, respectively. The *p* value from Student’s *t*-test between t8 and t9 is 0.81, suggesting that Bcd-dependent *hb* transcription has reached a background level that no longer changes over time. (**C**) Shown are the mean profiles of raw, background-subtracted *hb* intensities (in arbitrary units) detecting cytoplasmic mature mRNA at different time classes of cycle 14A. Here the adjacent T time classes differ by ∼5 min (see Fig. 2A). For clarity, only mean profiles are shown. See Fig. S4 for individual profiles. (**D**) Shown are *hb_plat_* values (in arbitrary units) detecting cytoplasmic mature mRNA in individual embryos from different time classes. The mean and standard deviation are: 24.03±6.37, 30.10±4.90, 33.78±5.88, 19.86±5.75 for T1 to T4, respectively.

To quantitatively evaluate our intron dot data, we calculated the mean intron dot number per nucleus, ρ, and plotted it as a function of fractional embryo length *x/L*. Figs. 4A–D show intron dot numbers in individual nuclei for representative embryos at t1, t3, t5 and t8, respectively, and Figs. 4E–H show their corresponding ρ profiles. These results show that ρ for the main *hb* domain declines quickly as a function of cycle 14A interphase progression (see Fig. 4D, H for a persistent PS4 stripe of intron dots). To better follow the dynamics of active *hb* transcription, we analyzed our intron dot data using embryos that are grouped into time classes (see above). Fig. 5A shows the mean ρ profiles as a function of *x/L* for time classes of t1 to t9 (see Fig. S3 for individual profiles). To further facilitate our analysis of Bcd-dependent active *hb* transcription near the P2 promoter, we calculated the mean intron dot number per nucleus at the plateau region (*ρ_plat_*) in individual embryos. The plateau region chosen for calculating *ρ_plat_* is devoid of the Bcd-independent *hb* stripe at PS4 [52], [53].

Fig. 5B is a plot of the calculated *ρ_plat_* values of cycle 14A embryos at different time classes. Our results show that the mean *ρ_plat_* rapidly increases immediately after entering the interphase, suggesting that the *hb* intron region near its P2 promoter resumes its active transcription status upon exiting mitosis (see below for further discussions). After reaching its peak at t3, the mean *ρ_plat_* begins to descend rapidly. The time at which the mean *ρ_plat_* drops to its half maximal level is between t4 and t5. By the time of t8, the mean *ρ_plat_* has dropped to background levels. Together, these results suggest that efficient *hb* transcription near its P2 promoter begins immediately upon entering the cycle 14A interphase, but it lasts only for a very short time, which is on the order of a few minutes. Our estimates suggest that by ∼5 min, *ρ_plat_* has dropped to its half maximal level, and by ∼8 min, *ρ_plat_* has dropped to background levels. This dynamic feature of active *hb* transcription near the P2 promoter contrasts with the relatively stable profile for mature *hb* mRNA as a function of time during cycle 14A interphase (see Figs 5C, S4 for mean and individual *hb* mRNA intensity profiles, respectively, in different time classes; and see Fig. 5D for *hb_plat_*, the mean *hb* mRNA intensity at the plateau region). These results show that monitoring intron dots can reveal dynamic properties of *hb* transcription at a temporal resolution that is much finer than monitoring mature *hb* mRNA or Hb protein.

### Prior to its Shutdown, Active *hb* Transcription Persists until the Moment when Nucleus Enters Mitosis

**Figure 6:**
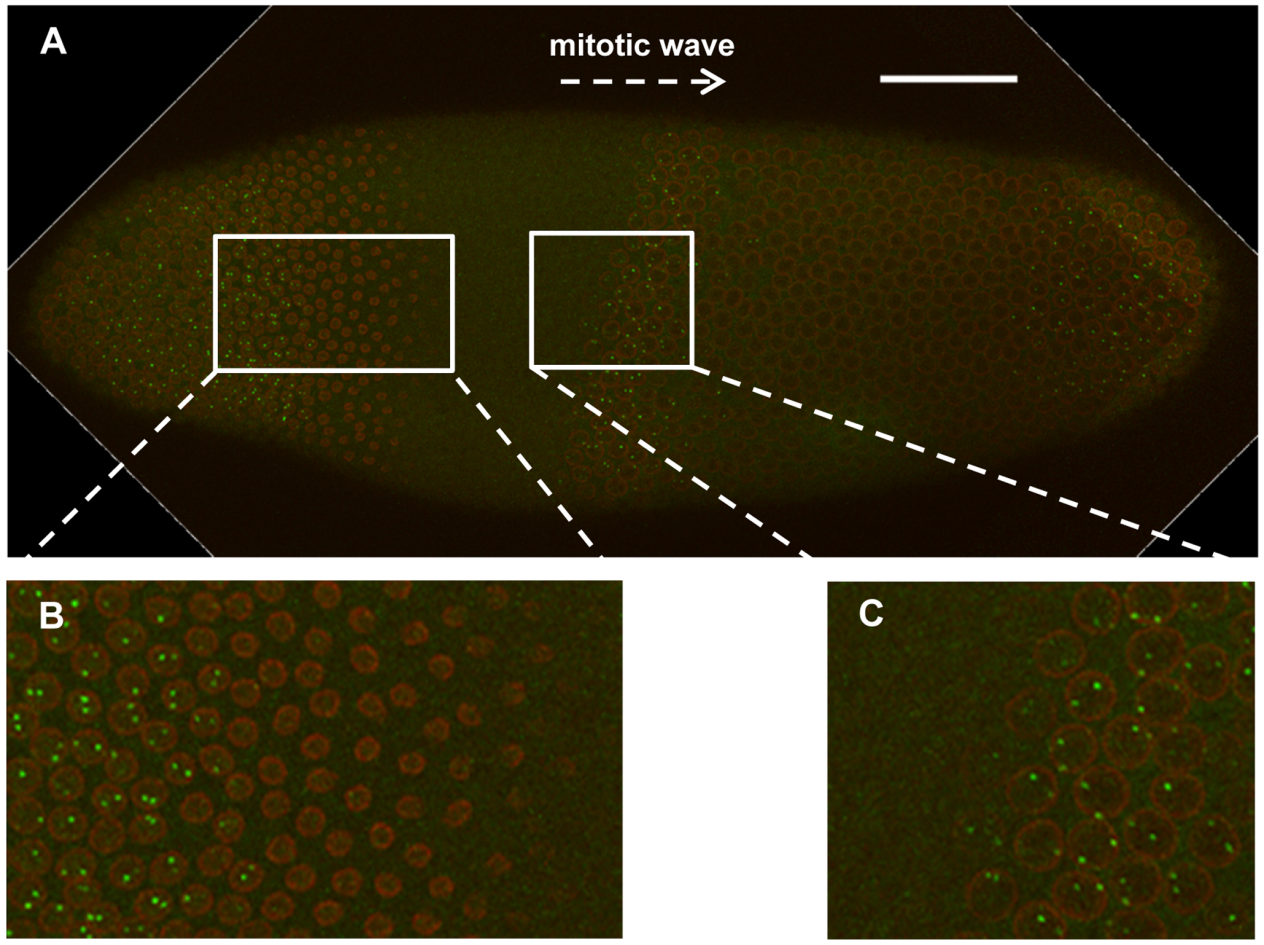
Active *hb* transcription at cycle 13 spans the entire interphase. (**A**) Shown is a merged confocal image of an embryo undergoing mitosis. This embryo has the 13^th^ mitotic wave on the anterior side, as evidenced by the breakdown of the nuclear envelope. Scale bar, 50 µm. (**B**) Shown is a higher magnification of a section of the image illustrating the reappearance of the cycle 14A nuclei and the *hb* intron dots (detected by an intronic probe) behind the mitotic wave. (**C**) Shown is a high magnification of a section of the image illustrating the disappearance of cycle 13 nuclear envelope and *hb* intron dots on the front of the mitotic wave.

Our observed dynamic behavior of active *hb* transcription at cycle 14A interphase contrasts with the transcription properties at earlier cycles. Unlike intron-stained embryos at cycle 14A, embryos at cycle 13 or 12 did not exhibit significant heterogeneity with regard to the presence of *hb* intron dots in the anterior (not shown). These results are consistent with a previous suggestion that embryos at earlier cycles are active in *hb* transcription throughout the entire interphases [26]. To further investigate the effect of mitosis on active *hb* transcription, we captured an embryo with the 13^th^ mitotic wave (where the nuclear envelope breaks down) sweeping through it (Fig. 6). Here, the *hb* intron dots were detectable immediately prior to the mitotic wave (Fig. 6C). Since our intron staining specifically detects nascent transcripts that contain the intron RNA sequence, our results suggest that active *hb* transcription near the P2 promoter was taking place until almost the very moment when the nucleus entered the mitotic phase. It is well established that mitosis can abort transcription [54]. In the embryo that we captured, *hb* intron dots also became detectable soon after the mitotic wave (Fig. 6B), indicating that, supportive of our quantitative data (Fig. 5B), active *hb* transcription near the P2 promoter resumed almost immediately upon entering the cycle 14A interphase. These results, together with those described previously [26], suggest that, up until the onset of the cycle 14A interphase, efficient *hb* transcription initiation/elongation near the Bcd-responsive P2 promoter spans the entire interphase durations. This dynamics of active *hb* transcription in embryos at earlier interphases is in sharp contrast with that in embryos at cycle 14A interphase.

The image shown in Fig. 6 makes it possible to have some estimates of active *hb* transcription in relation to the exit of mitosis at a finer time scale. Based on the estimated speed of the mitotic wave in early embryos [47], the time between the appearance of the nuclear envelope and appearance of the first *hb* intron dots is likely to be measured on the scale of seconds. The time it takes RNAP to travel from the P2 promoter to its first intron (located 145 bp downstream) is expected to be on a similar time scale [51], [54], [55]. According to these estimates, our results suggest that RNAP likely begins to transcribe the *hb* gene from the P2 promoter almost simultaneously as the nuclei are exiting mitosis. It remains unknown how RNAP can resume active *hb* transcription at the P2 promoter so quickly after mitosis, a question relevant to another fundamental question of how the positional information provided by the Bcd gradient could be decoded to produce reliable gene expression boundaries at a time when the embryo is undergoing rapid cycles of nuclear division [56]. We note that, since our discussions here are based on an embryo (Fig. 6) that we were able to capture as an example, more systematic investigations are required to draw more quantitative conclusions about the precise relationship between mitosis and *hb* transcription.

### Nuclear Bcd Profile and Persistent Transcription of Another Gap Gene

**Figure 7:**
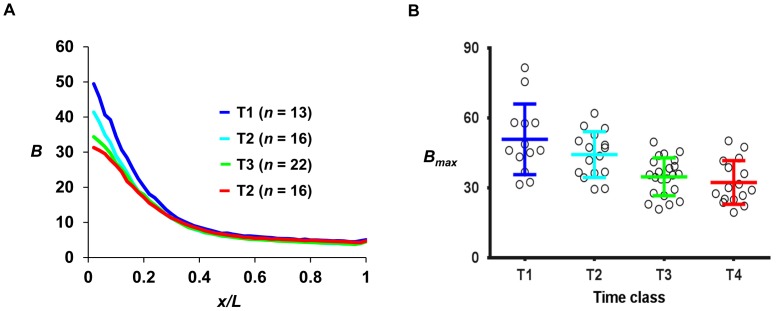
Bcd molecules are abundant at the time of *hb* transcription shutdown. (**A**) Shown are the mean profiles of raw Bcd intensities (in arbitrary units) from cycle 14A embryos at different time classes. Each color represents a time class; the adjacent T time classes differ by ∼5 min (see Fig. 2A). For clarity, only mean profiles are shown. See Fig. S5 for individual profiles. (**B**) Shown are raw *B_max_* values (in arbitrary units) for individual embryos from different time classes T. The mean and standard deviation are: 50.92±15.15, 44.39±9.83, 35.08±8.16, 32.12±9.22 for T1 to T4, respectively.

The dynamics of *hb* transcription shutdown raises a question about the dynamic changes in the nuclear concentration of Bcd at the time of *hb* shutdown. Previous studies [39], [57] suggest that the profiles of nuclear Bcd concentration should remain intact in embryos at the time of *hb* shutdown. To further investigate this issue under our current experimental and analytical framework, we performed quantitative fluorescent immunostaining experiments to detect Bcd in embryos that are grouped into appropriate time classes. Fig. 7A shows the mean profiles of Bcd fluorescent intensities in embryos of time classes T1–T4 (see Fig. S5 for profiles of individual embryos). T time classes were used in this analysis because the Bcd gradient profiles are relatively stable in comparison with the dynamics of active *hb* transcription. Our results (Fig. 7B) are consistent with the reported live-imaging studies with regard to the evolution of nuclear Bcd intensities [57]. They show that, although the maximal Bcd intensities (*B_max_*) exhibit a steady decrease during the cycle 14A interphase, the Bcd gradient profile remains largely intact at or even after the time of *hb* shutdown (which corresponds to T2; see Fig. 1A for a schematic diagram depicting the relationship between t and T time classes). These results suggest that the nuclei in the anterior part of the embryo continue to have abundant nuclear Bcd available for transcription activation well after the time of *hb* shutdown.

**Figure 8:**
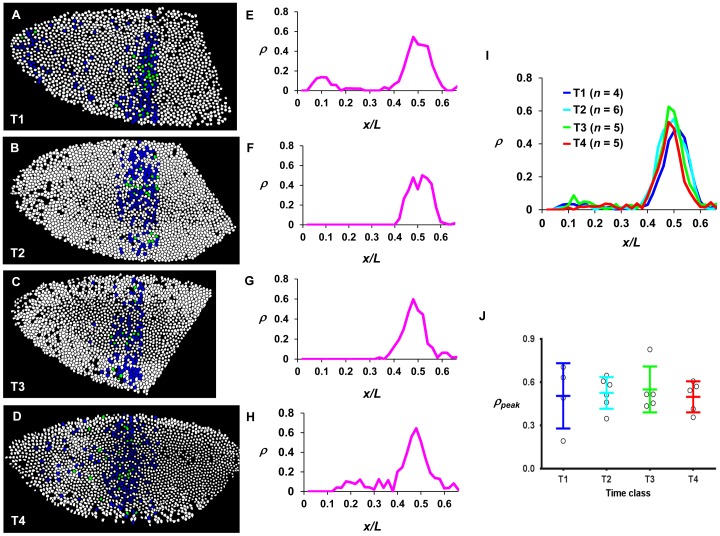
Quantitative analysis of active *Kr* transcription at cycle 14A. (**A–D**) Shown are diagrams to illustrate the status of active *Kr* transcription for individual nuclei in embryos at T1 to T4, respectively. See Fig. 4 legend for color code. Here nascent *Kr* transcripts were detected by an intronic probe. (**E–H**) Shown are ρ profiles as a function of *x/L* from panels **A–D**, respectively. (**I**) Shown are the mean *ρ* profiles as a function of *x/L* at early nuclear cycle 14A. The adjacent T time classes differ by ∼5 min (see Fig. 2A). (**J**) Shown are *ρ_peak_* values for individual embryos in different T time classes. The mean and standard deviation are: 0.51±0.23, 0.53±0.11, 0.55±0.16, 0.50±0.11 for T1 to T4, respectively. There is no significant difference between any pairs of two values according to Student’s *t*-tests.

It is well documented that gap genes undergo extensive cross-regulation as cycle 14A progresses [4], [5], [40]. Genes that are sensitive to cross-regulation at the level of transcription must be transcriptionally active to maintain their responsiveness to such inputs. To experimentally investigate this issue, we performed an intron staining analysis for *Kr*, which is responsive to inputs of gap gene products and likely the Bcd gradient as well [58]–[61]. Like the *hb* intron, the *Kr* intron is also short (373 bp) and located relatively close to its promoter (374 bp downstream; [62]). Thus *Kr* intron dots, similar to *hb* intron dots, also monitor closely (but still not exactly) the *Kr* transcription initiation/elongation near the promoter. Fig. S6 shows a surface image of a cycle 14A embryo with the detected *Kr* intron dots forming a central stripe that resembles the *Kr* mRNA pattern, documenting the sensitivity of the probe used in our analysis. We analyzed our *Kr* intron dot data in the same way as for *hb* intron dot data. Since the *Kr* intron dot profile persists for much longer time (see below), T time classes were used in this analysis. Figs. 8A–H shows typical ρ profiles for *Kr* as a function of *x/L* from individual embryos at time classes of T1 to T4. Fig. 8I shows the mean ρ profiles for different time classes (see Fig. S7 for individual profiles). To facilitate further comparison, the mean *ρ* at the peak region *(ρ_peak_*) of *Kr* intron dots in individual embryos were calculated and plotted for different time classes (Fig. 8J). These results show that the mean *ρ_peak_* is largely similar among the time classes analyzed, demonstrating that, unlike *hb*, active *Kr* transcription persists for at least ∼20 min into the cycle 14A interphase. They provide a clear documentation that two gap genes within the AP patterning network, *hb* and *Kr*, exhibit distinct dynamic profiles of active transcription at cycle 14A.

## Discussion

Our study uncovers a dynamic feature of the AP patterning network in *Drosophila* at a time–early cycle 14A–during which the cross-regulatory mechanisms begin to intensify. The quick shutdown of *hb* transcription in the Bcd-dependent primary domain contrasts with *Kr* transcription (Fig. 8), which is known to be subject to extensive cross-regulation by gap genes [58]–[61]. This shutdown also contrasts with *hb* transcription at PS4 (Figs. 4D, H, and 5A), which is also known to respond to cross-regulatory mechanisms during cycle 14A [24], [41]. While the PS4 and Bcd-dependent domains have overlapping posterior boundaries, they have distinct transcriptional dynamics and respond to distinct regulatory inputs. When Bcd-dependent *hb* transcription is already shut down and becomes transcriptionally non-responsive, Bcd-independent *hb* transcription at PS4 persists. This dynamic difference between these two *hb* expression domains represents an important feature of the AP patterning network, which provides a fundamental explanation for why cross-regulatory mechanisms for *hb* expression appear to operate primarily through its expression at PS4 [24], [41].

The inputs for *hb* PS4 expression at cycle 14A include both the Bcd-driven Hb and two other gap gene products Kr and Kni [24], [41]. The quick shutdown of the Bcd-dependent *hb* transcription at early cycle 14A (which renders it transcriptionally non-responsive) has a powerful impact on the regulatory logic within the AP patterning network. It is suggested that the PS4 transcription is autoactivated by Hb [31], [34]. Thus, the dynamic difference in active transcription between these two domains should enable the product of Bcd-dependent *hb* transcription to have a significant impact on the expression at PS4, but not the other way around. We suggest that our observed shutdown of *hb* transcription at the primary domain represents a design feature of the AP pattern network to allow *hb* to directly benefit from the scaling properties of the Bcd gradient. Specifically, the Bcd gradient profiles in large embryos can “reach” farther into the posterior so that *hb* expression boundary position can become scaled with the embryo length [15], [17], [63]. Importantly, having a boundary position near mid embryo, the role of *hb* in regulating the expression of other gap genes is well documented [27], [61], [64]. It should be noted that, for genes that respond to Hb protein as an input, it is the integrated Hb level (derived from both expression domains) that is instructive to their expression patterns in the embryo. Thus the transition from the primary to secondary *hb* domains represents a network design that allows *hb* to directly and faithfully respond to the Bcd input while still enabling Hb protein to fully (with regard to time) participate in the cross-regulatory network to impact the entire landscape of AP patterning. A gene expression pattern boundary that is composed of two (or more) spatially and temporally overlapping boundaries can be viewed as a montage boundary. For the observed montage *hb* boundary near mid embryo at cycle 14A, the spatial overlap is reflective of the operation of the distinct inputs that act on distinct enhancers [24], [31]. The temporal overlap is reflective of the perdurance of the *hb* gene products (mature mRNA and protein) despite a quick shutdown of Bcd-dependent active transcription.

The scaling properties of Bcd are proposed to be derived from an egg volume-dependent maternal deposition of *bcd* mRNA [15], suggesting that a robust and scaled AP patterning outcome of the embryo may depend on events that go as far back in time as oogenesis. While cross-regulatory mechanisms can achieve scaling and precision on their own [6], the degree of *hb* precision and scaling that can be derived directly from the Bcd gradient early on will affect the intensity and contribution of subsequent cross-regulatory mechanisms necessary for patterning precision near mid embryo where the *hb* boundary is located. The *hb* transcriptional dynamics documented in our current work should thus be beneficial to future modeling studies of developmental robustness. We note that our current study focuses on *hb* expression at the level of active transcription and, thus, not informative to regulatory mechanisms at other levels. Going beyond AP patterning in *Drosophila*, our study underscores the general importance, in developmental biology, of dissecting the distinct events that may create a montage of an expression boundary (or a montage of an expression pattern) that we observe.

Prior to its shutdown at early cycle 14A interphase, active *hb* transcription spans the entire interphases (Fig. 6; [26]). In addition, prior to cycle 13, the probability of Bcd-dependent active *hb* transcription is further boosted by maternal Hb [26]. Both of these features of *hb* transcription benefit the accumulation of Bcd-dependent products at a time when the number of nuclei (i.e., the *hb* gene template number available for producing *hb* mRNA and Hb protein in the embryo) was low but undergoing an exponential increase after each nuclear cycle. Importantly, maternal Hb does not have a significant effect on the boundary position of the primary *hb* domain [26]. It is currently not known mechanistically how maternal Hb specifically regulates the Bcd-dependent output level without conferring significant positional information to modulate the Bcd-dependent *hb* expression boundary. As a DNA-binding transcription factor, Hb is known to have the ability to provide positional information to regulate gene expression patterns in a concentration-dependent manner [27], [64]. It remains to be elucidated whether the special properties of the maternal Hb on Bcd-dependent *hb* expression has a role in developmental robustness.

Precisely how *hb* transcription is shut down in early cycle 14A embryos remains unknown at this time. The shutdown appears to be position-independent (Fig. S8). The Bcd gradient profile remains largely intact at (and after) the time of *hb* shutdown (Fig. 7; [57]). Were the shutdown merely due to a decrease in nuclear Bcd concentration, one might have expected a position-dependent shutdown, “initiating” from regions of the embryo with low Bcd concentrations and “spreading” into regions with higher Bcd concentrations. But our results did not indicate such a position-dependent shutdown (Fig. S8). A position-independent, uniform shutdown of *hb* transcription at early cycle 14A also suggests that, if “conventional” repressors (e.g., DNA-binding proteins encoded by gap genes) are responsible for this shutdown, there would need to be a sufficient number of them to cover all the locations (where the primary *hb* expression domain is located) and they would all need to cross synchronously their respective concentration thresholds for *hb* repression. Further studies are required to identify the players and mechanisms for *hb* shutdown at early cycle 14A. We note that, *sex-lethal*, which plays a critical role in the fundamental biological process of dosage compensation in *Drosophila*, also exhibits a quick shutdown, at a time similar to that of *hb* shutdown [65]. Metazoan embryos share a common feature in that they start with a series of rapid, synchronous cell divisions, followed by a sudden slowing in division and onset of asynchronous divisions and morphogenic movements [66]–[68]. This period is referred to as the mid-blastula transition (MBT), which corresponds to the cycle 14A interphase in *Drosophila* [66]–[68]. MBT coincides with a major shift in the embryo’s RNA contents, from maternal RNA to the onset of bulk transcription from the zygotic genome, referred to as the maternal-to-zygotic transition (MZT; [69]). While mechanisms that promote early zygotic transcription have attracted significant recent interests [70]–[72], mechanisms for proper shutdown of some early-expressing genes with key regulatory functions, such as *hb* and *sex-lethal*, may also be integral to MZT.

While our work was under consideration for publication, a study by Surkova et al. was published [73]. The findings reported in that paper are consistent with our suggestion that the gap gene cross-regulatory mechanisms for *hb* transcription operate through the PS4 expression domain. In particular, mutations that lead to increased variation in the *hb* boundary (at later times of cycle 14A) are those that disrupt the formation of the *hb* PS4 domain. Furthermore it is documented that none of the gap gene mutations tested caused a shift in the *hb* boundary position. Those results, together with our results described in the current report, are consistent with a model where 1) the mean position of the montage *hb* boundary is determined primarily by the Bcd input and 2) the precision of this montage boundary depends on the initial action of Bcd on regulating the primary *hb* domain and, later in time, is further corrected (for the remainder errors) by cross-regulatory mechanisms that act on the PS4 domain. This model can explain the reported findings including our own observations that alterations in Bcd gradient properties cause direct and corresponding alterations in the *hb* expression boundary with regard to both position and precision [17], [28], [36]–[38].

## Supporting Information

Figure S1 **Time classification of cycle 14 embryos.** Shown are nuclear heights (µm) of individual embryos in different t time classes (mean and standard deviation are shown) for the analysis of *hb* transcription dynamics shown in Figs. 5A and 5B. See Fig. 2A for a schematic diagram showing the estimated locations of these time classes in relation to other time events at cycle 14A.
(TIF)

Figure S2 **Mature** ***hb*** **mRNA pattern at early cycle 14A.** Shown are midsegittal images from embryos at time classes T1 (A and B), T2 (C and D), T3 (E and F), and T4 (G and H), respectively. Panels A, C, E and G show the *hb* mRNA signals from FISH experiments. Panels B, D, F and H show the corresponding embryos with nuclear staining.
(TIF)

Figure S3 ***ρ*** **profiles of** ***hb*** **extracted from individual embryos.** Data at time classes t1 to t9 are shown in (A–I), respectively. Each color represents data from an individual embryo.
(TIF)

Figure S4 ***hb*** **mRNA intensity profiles extracted from individual embryos.** Data at time classes T1–T4 are shown in (A–D), respectively. The mean intensities (in arbitrary units) and error bars (standard deviation) are shown.
(TIF)

Figure S5 **Bcd intensity profiles extracted from individual embryos.** Bcd intensity data (in arbitrary units) detected in embryos at time classes T1–T4 are shown in (A–D), respectively.
(TIF)

Figure S6 **Intron staining detecting nascent transcripts near the** ***Kr*** **promoter.** (A) Shown is a merged confocal image of an embryo at cycle 14A. (B) is a magnified view of a section of the expression region from panel A. The detected nuclear envelope is shown in red and the nascent *Kr* transcripts detected (with an intronic probe) as intron dots are in green. Scale bar, 50 µm.
(TIF)

Figure S7 ***ρ*** **profiles of** ***Kr*** **extracted from individual embryos.** Data at time classes T1 to T4 are shown in (A–D), respectively. These profiles were from intron staining with a *Kr*-specific intronic probe.
(TIF)

Figure S8 **Position-independent** ***hb*** **shutdown at the plateau region.** Show are time evolution profiles of the mean ρ values from each of the five positions at the plateau region of *hb* expression, with Bin #1 denoting the most anterior position. The averaged profile from all the five bins, which is shown in Fig. 5B, is also shown here for reference.
(TIF)
