# Correction: Uncovering a Dynamic Feature of the Transcriptional Regulatory Network for Anterior-Posterior Patterning in the *Drosophila* Embryo

**DOI:** 10.1371/annotation/32c2ee82-b92f-4984-ab58-5cccdebaf8e3

**Published:** 2014-01-17

**Authors:** Junbo Liu, Jun Ma

In the Supporting Information, Figure S8 is incorrect. The correct version of Figure S8 can be found here: http://plosone.org/corrections/ppone.0062641.s008.cn.pdf

