# Correction: Uncovering a Dynamic Feature of the Transcriptional Regulatory Network for Anterior-Posterior Patterning in the *Drosophila* Embryo

**DOI:** 10.1371/annotation/30720b12-d726-4467-b89c-dcd7b5367855

**Published:** 2014-01-17

**Authors:** Junbo Liu, Jun Ma

Please see the correct version of Figure S8 here: http://plosone.org/corrections/pone.0062641.s008.cn.pdf

