# Supplementary figures and images for: Uncovering a Dynamic Feature of the Transcriptional Regulatory Network for Anterior-Posterior Patterning in the Drosophila Embryo
Source: PLoS One. 2013 Apr 30;8(4):e62641. doi: 10.1371/journal.pone.0062641 (PMC3639989; doi:10.1371/journal.pone.0062641)

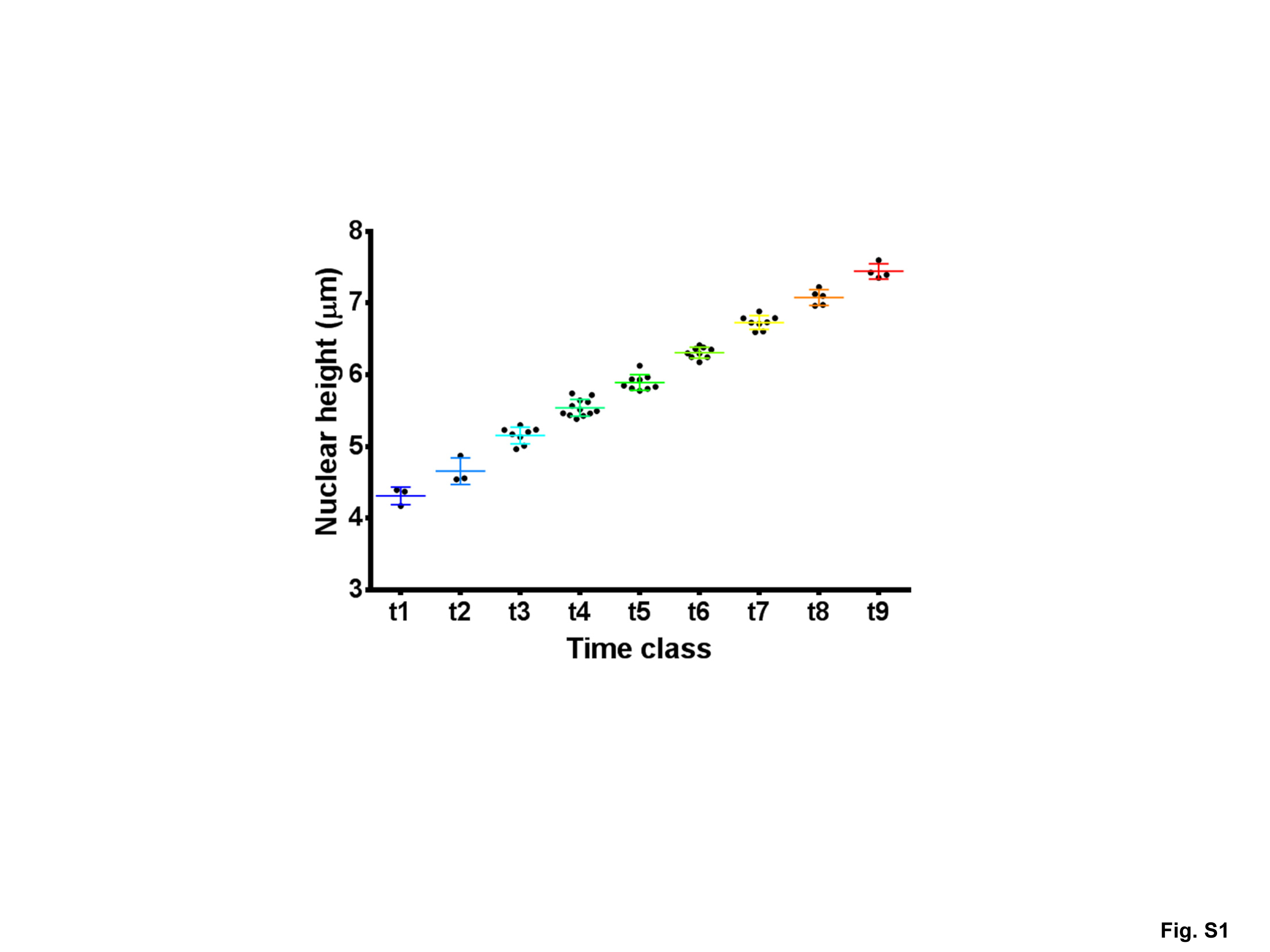

Supplement: Figure S1 — Time classification of cycle 14 embryos. Shown are nuclear heights (µm) of individual embryos in different t time classes (mean and standard deviation are shown) for the analysis of hb transcription dynamics shown in Figs. 5A and 5B. See Fig. 2A for a schematic diagram showing the estimated locations of these time classes in relation to other time events at cycle 14A. (TIF) [file pone.0062641.s001.tif]

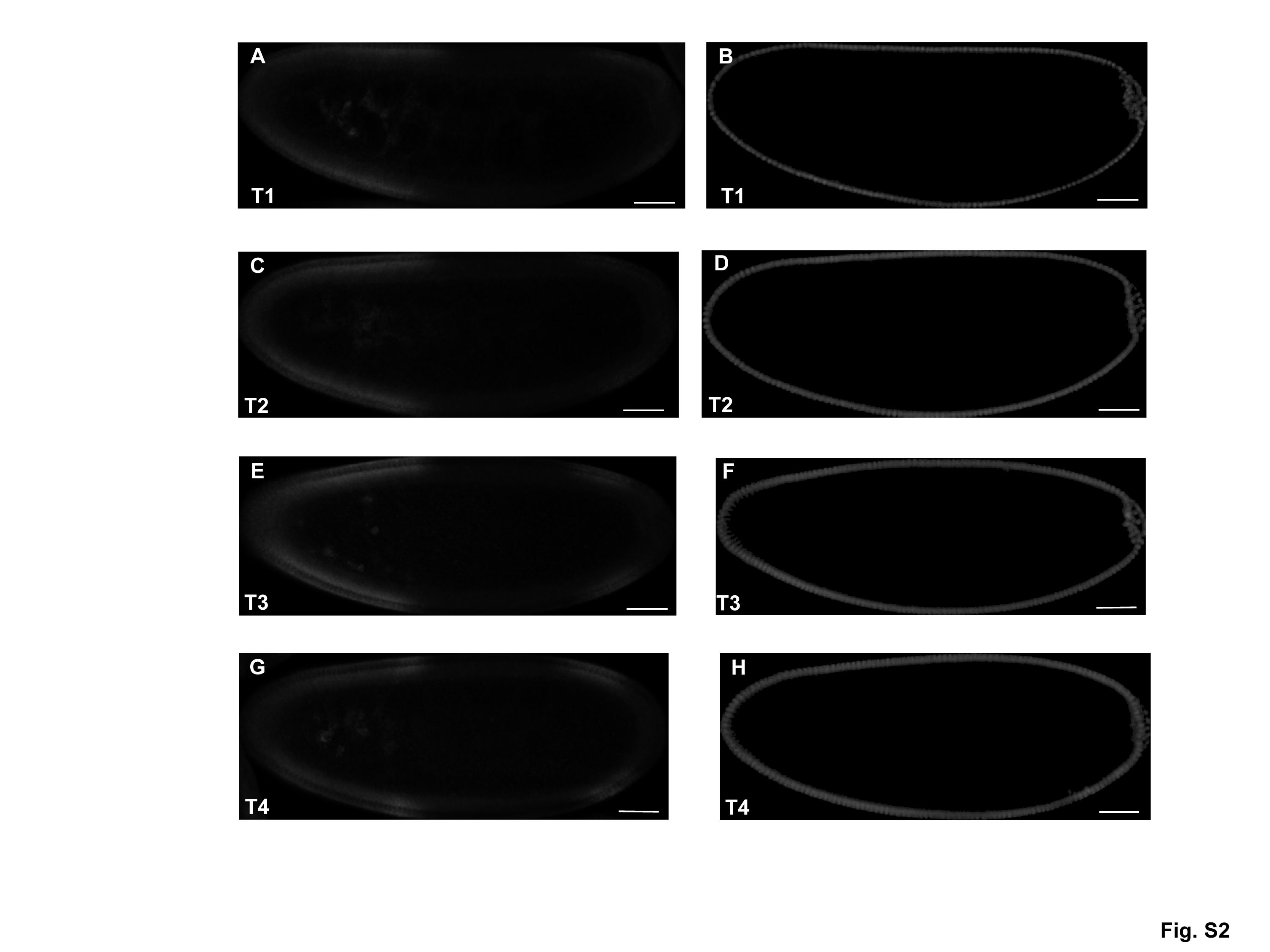

Supplement: Figure S2 — Mature hb mRNA pattern at early cycle 14A. Shown are midsegittal images from embryos at time classes T1 (A and B), T2 (C and D), T3 (E and F), and T4 (G and H), respectively. Panels A, C, E and G show the hb mRNA signals from FISH experiments. Panels B, D, F and H show the corresponding embryos with nuclear staining. (TIF) [file pone.0062641.s002.tif]

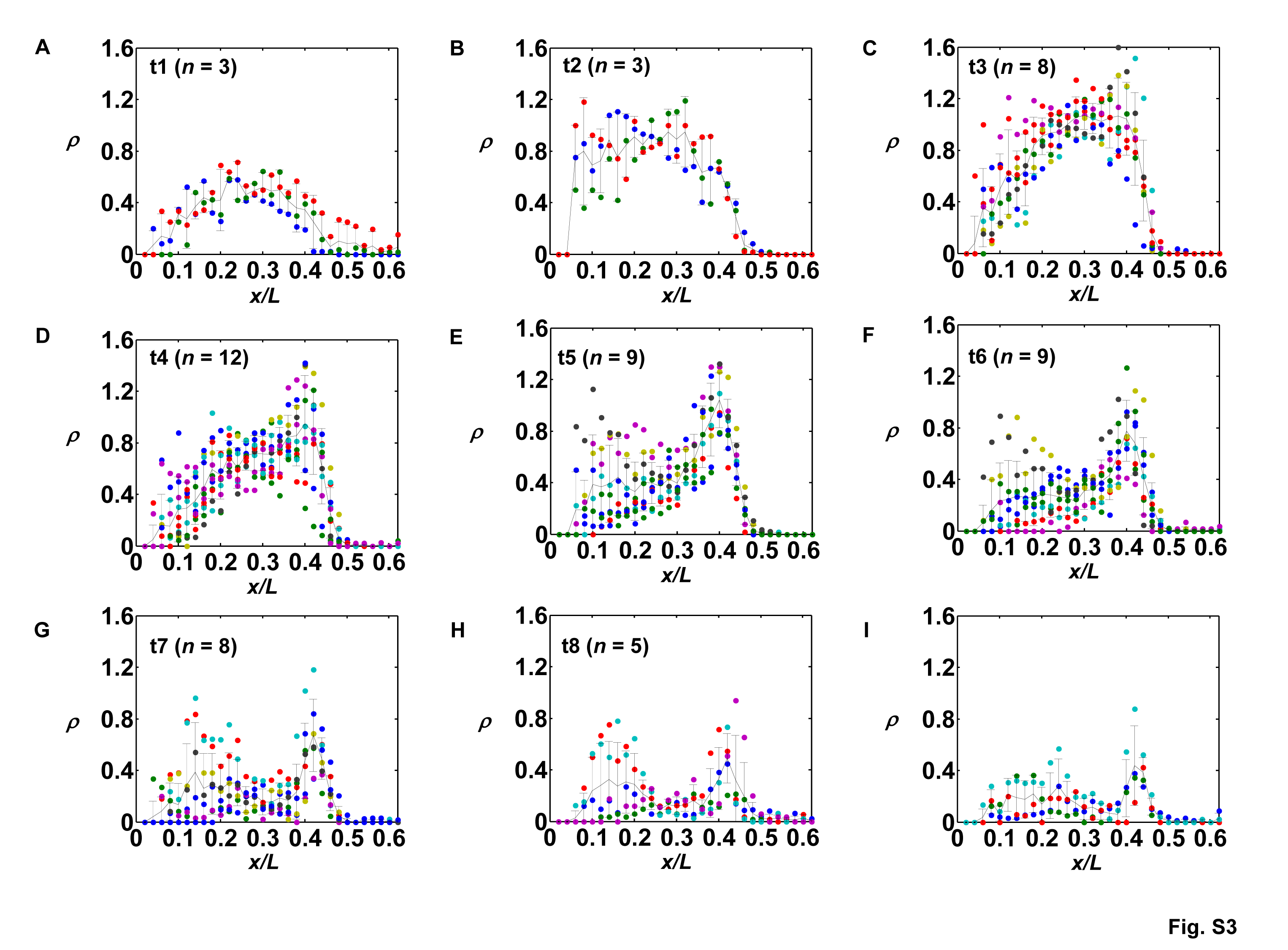

Supplement: Figure S3 — ρ profiles of hb extracted from individual embryos. Data at time classes t1 to t9 are shown in (A–I), respectively. Each color represents data from an individual embryo. (TIF) [file pone.0062641.s003.tif]

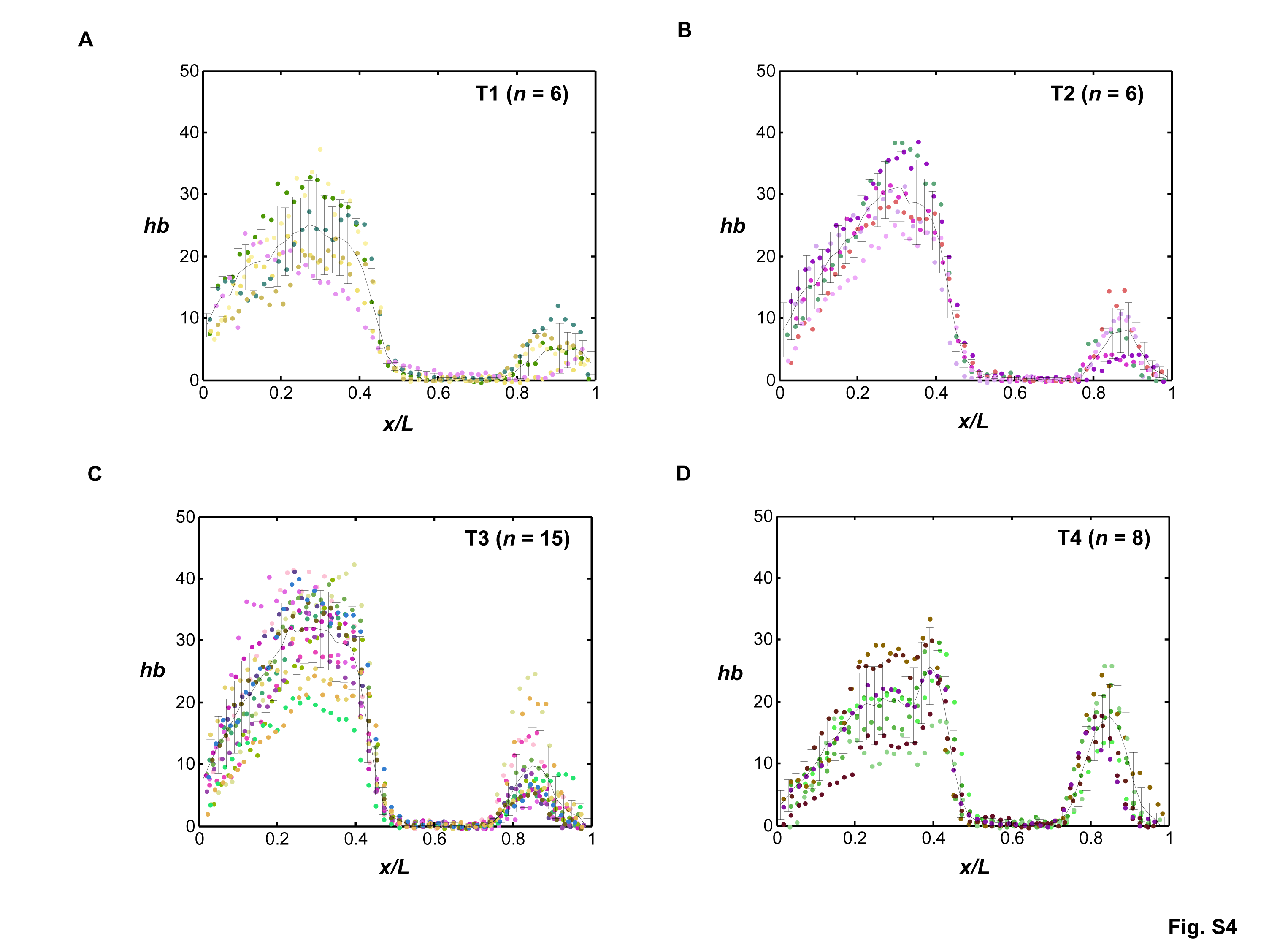

Supplement: Figure S4 — hb mRNA intensity profiles extracted from individual embryos. Data at time classes T1–T4 are shown in (A–D), respectively. The mean intensities (in arbitrary units) and error bars (standard deviation) are shown. (TIF) [file pone.0062641.s004.tif]

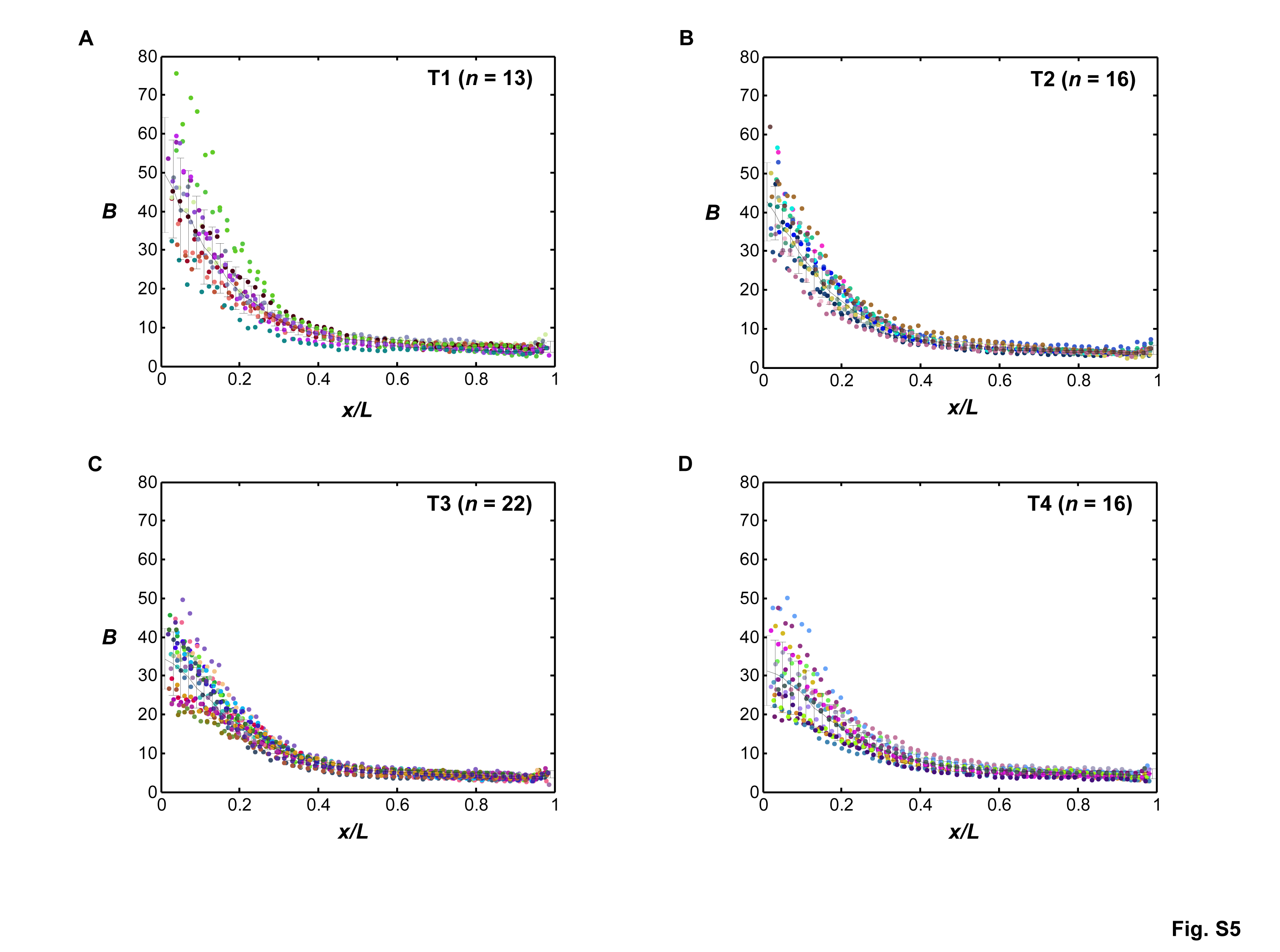

Supplement: Figure S5 — Bcd intensity profiles extracted from individual embryos. Bcd intensity data (in arbitrary units) detected in embryos at time classes T1–T4 are shown in (A–D), respectively. (TIF) [file pone.0062641.s005.tif]

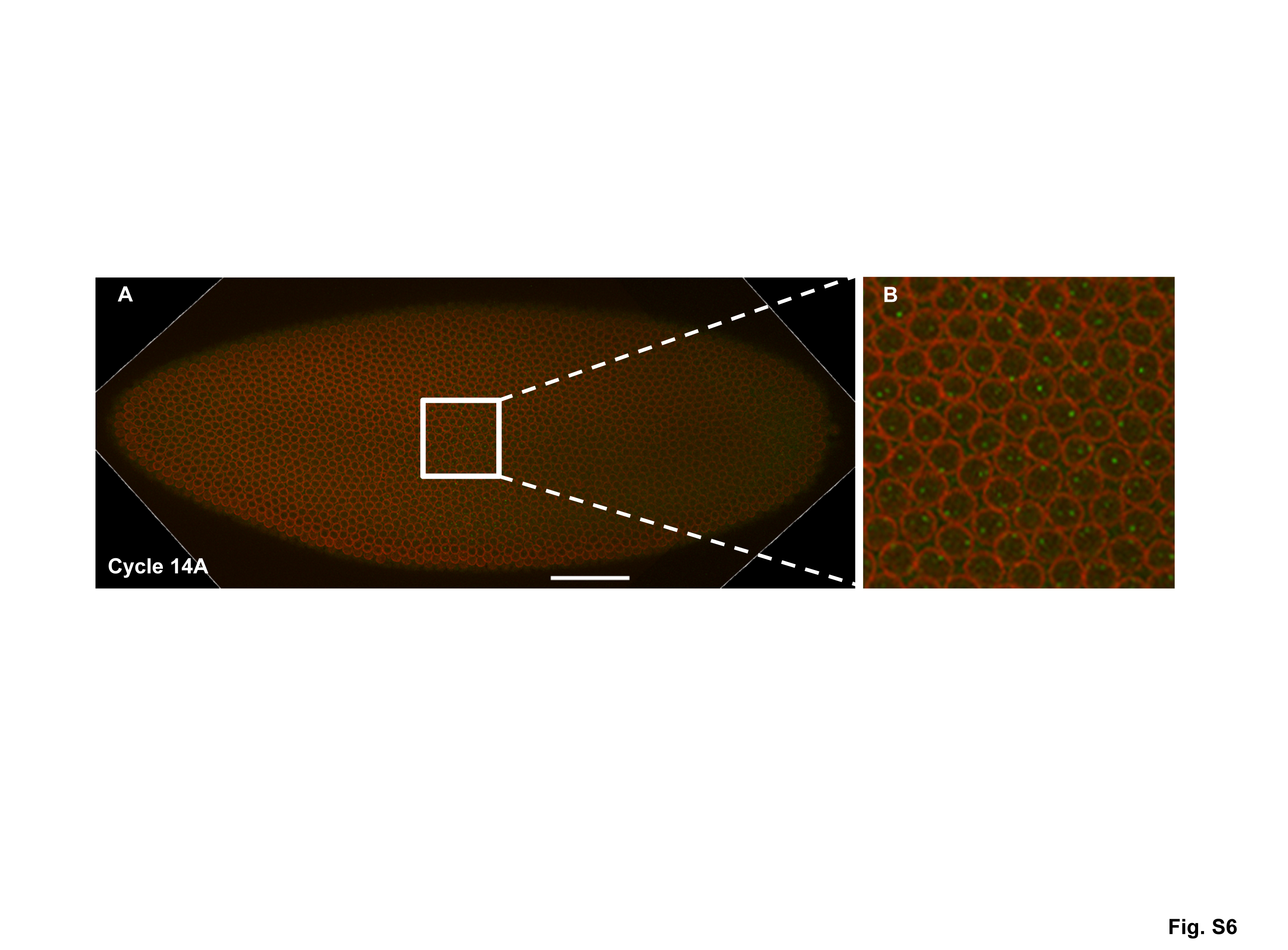

Supplement: Figure S6 — Intron staining detecting nascent transcripts near the Kr promoter. (A) Shown is a merged confocal image of an embryo at cycle 14A. (B) is a magnified view of a section of the expression region from panel A. The detected nuclear envelope is shown in red and the nascent Kr transcripts detected (with an intronic probe) as intron dots are in green. Scale bar, 50 µm. (TIF) [file pone.0062641.s006.tif]

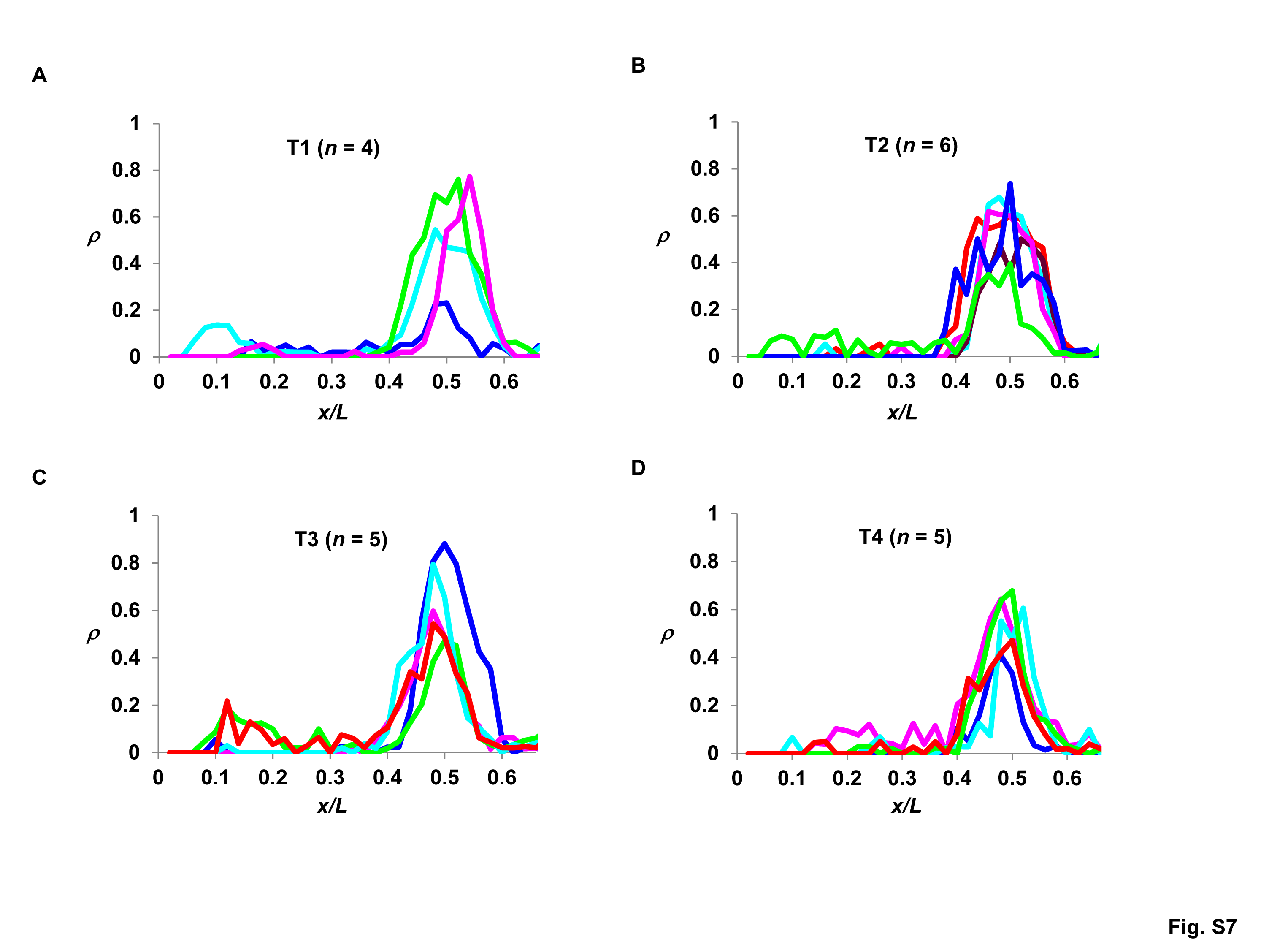

Supplement: Figure S7 — ρ profiles of Kr extracted from individual embryos. Data at time classes T1 to T4 are shown in (A–D), respectively. These profiles were from intron staining with a Kr-specific intronic probe. (TIF) [file pone.0062641.s007.tif]

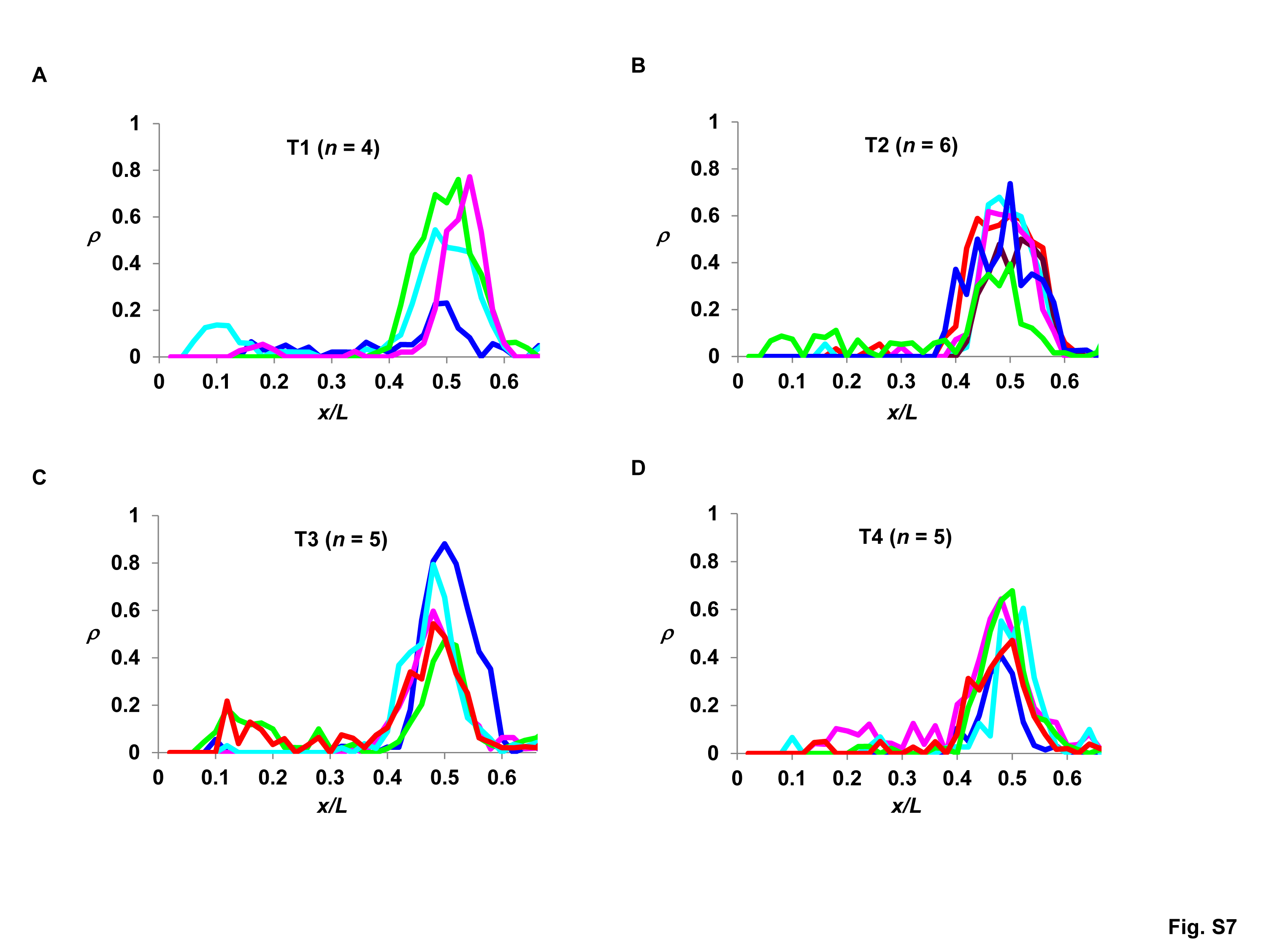

Supplement: Figure S8 — Position-independent hb shutdown at the plateau region. Show are time evolution profiles of the mean ρ values from each of the five positions at the plateau region of hb expression, with Bin #1 denoting the most anterior position. The averaged profile from all the five bins, which is shown in Fig. 5B, is also shown here for reference. (TIF) [file pone.0062641.s008.tif]
